# Genome-Wide Characterization of the *WOX* Gene Family and Identification of Key *PmWOX4* Gene Associated with Adventitious Root Formation in *Pinus massoniana*

**DOI:** 10.3390/plants15121845

**Published:** 2026-06-15

**Authors:** Wenjuan Su, Boyuan Fan, Jing Liu, Sheng Yao, Xiang Cheng, Zhikui Wang, Kongshu Ji

**Affiliations:** 1 State Key Laboratory of Tree Genetics and Breeding, Nanjing Forestry University, Nanjing 210037, China; suwenj@njfu.edu.cn (W.S.); byfan@njfu.edu.cn (B.F.); 18130036897@163.com (J.L.); yaosheng0817@njfu.edu.cn (S.Y.); chengxiang@njfu.edu.cn (X.C.); wzk041104@163.com (Z.W.); 2 Co-Innovation Center for Sustainable Forestry in Southern China, Nanjing Forestry University, Nanjing 210037, China

**Keywords:** *Pinus massoniana*, *WOX* gene family, adventitious root, expression pattern

## Abstract

*Pinus massoniana* Lamb. is an important native economic and ecological tree species in southern China. However, adventitious root regeneration severely restricts the asexual propagation of elite traits. The *WOX* gene family, a plant-specific transcription factor family, plays critical roles in various aspects of plant growth and development, particularly in root development, including the maintenance of the root apical meristem, root elongation, and the initiation and formation of lateral roots. In this study, a total of 21 WOX family proteins were identified from the genome of *P. massoniana* and designated as *PmWOX1* to *PmWOX21* based on their chromosomal locations. These 21 members were unevenly distributed across nine chromosomes, with a clustered distribution observed on chr7. Collinearity analysis suggested that gene duplication and purifying selection may serve as key driving forces in the evolution of *WOX* genes. Conserved motif analysis revealed divergence among different PmWOX clades, implying distinct functions in growth and development. Cis-element analysis indicated that *PmWOX* genes may be involved in the regulation of stress responses, hormone signaling, and developmental processes. Expression pattern analysis based on transcriptome data from root tips and shoot tips identified nine *PmWOX* genes with significantly higher expression in root tips. RT-qPCR further screened *PmWOX4*, which exhibited the highest expression in root primordia. Functional experiments verified that *PmWOX4* genes encoded nuclear-localized proteins with transcriptional activity. The PPI results indicate that PmWOX4 may interact with CLE41/44, LBD, ATHB8 and CLV3/CLV1 to regulate adventitious root formation. Collectively, our findings suggest that *PmWOX4* possesses functional potential in adventitious rooting, which could be further exploited to improve the efficiency of asexual propagation in *P. massoniana*.

## 1. Introduction

*Pinus massoniana* Lamb., a member of the Pinaceae family and the *Pinus* genus, is a highly valuable native resin-producing tree species in China, widely distributed across the country [1,2]. This species exhibits strong adaptability, rapid growth, and excellent productivity, making it a cornerstone for afforestation and the development of the forestry industry in southern China, with significant ecological and economic value [3]. However, the increasing scarcity of superior provenances and considerable fluctuation in seed orchard yields indicate that sexual reproduction alone cannot meet the demand for high-quality seedlings required for large-scale afforestation [4]. In comparison to conventional sexual reproduction, the vegetative propagation of *P. massoniana* effectively preserves the superior genetic traits of elite individuals, thereby avoiding issues such as segregation in offspring, unstable seed production, and prolonged nursery periods. This method serves as a core technique for conserving superior germplasm, scaling up the multiplication of improved varieties, and promoting their industrial application [5]. Common problems in the asexual propagation of *Pinus* species include explant browning, poor differentiation, and difficulties in rooting. Among these, the extremely low efficiency of adventitious root induction and the challenges associated with rooting are the primary obstacles hindering the asexual propagation of *P. massoniana,* significantly contributing to its notably low success rate in tissue culture regeneration [6]. Therefore, overcoming rooting difficulties and enhancing rooting efficiency in *P. massoniana* tissue culture are of great theoretical and practical value for addressing the core technical limitations of its rapid clonal propagation, improving the vegetative propagation system, and facilitating the efficient use of elite germplasm in large-scale afforestation.

The *WOX* genes represent a family of plant-specific transcription factors that play critical roles in plant development and stress responses [7]. They are involved in essential processes such as embryonic development, stem cell maintenance, organ formation, and the induction of somatic embryogenesis [8,9]. A defining feature of *WOX* transcription factors is the presence of a 60-amino-acid homeodomain (HD). WUS, the first identified member of the WOX family, contains an additional tyrosine (Y) residue compared to other WOX members and adopts a “helix–loop–helix–turn–helix” structure [10]. Notably, the “helix–turn–helix” motif formed by the second and third helices is capable of binding to specific DNA sequences, thereby regulating the transcription of downstream genes [11].

Numerous studies have demonstrated that members of the *WOX* gene family play indispensable regulatory roles in maintaining root meristems, promoting root elongation, and initiating lateral root formation. In apple (*Malus domestica*) rootstocks, the heterologous overexpression of the *MdWOX4B* gene significantly enhances adventitious root formation [12]. In *Populus tomentosa*, *PtoWOX4a* is primarily expressed in the root stele, root tip regions, and the vascular tissues of leaves and stems. Overexpression of *PtoWOX4a* enhances adventitious root regeneration and increases the number of adventitious roots, although it inhibits their length [13]. Overexpression of *PtoWOX5a* results in significant phenotypic changes in adventitious root development from both roots and leaves, characterized by an increased number of adventitious roots, reduced length, and swelling of adventitious root tips and lateral root apices [14]. In the study of *WOX* transcription factors in ‘Nanlin895’ (*Populus* × *euramericana* ‘Nanlin895’), it was found that *PeWOX11a* and *PeWOX11b* are involved in multiple developmental processes in poplar, particularly in adventitious root formation. Overexpression of either *PeWOX11a* or *PeWOX11b* not only increases the number of adventitious roots on cuttings but also induces ectopic root formation in the aerial parts of transgenic poplars [15]. In recent years, several studies have also been conducted on gymnosperm conifers. In *Picea abies*, *PaWOX4* is expressed across all tissues, with the highest expression levels observed in shoot tips, cambium, and root tips. Interestingly, its levels are greater in the hypocotyls of seedlings, whereas they are diminished in shoot tips, cotyledons, and root tips [16]. Additionally, the overexpression of *Larix gmelinii* leads to a marked increase in the count of adventitious roots, but it also causes a decrease in root length in 84K poplar [17].

This study research conducted a comprehensive identification and systematic analysis of the *WOX* gene family in *P. massoniana* across the entire genome. The study examined various aspects, including the physicochemical properties, distribution on chromosomes, phylogenetic relationships, gene structure, and conserved motifs associated with the *WOX* genes. Alongside the prediction of cis-acting elements in promoters and an analysis of expression pattern across different tissues, the evolutionary traits of the *PmWOX* gene family and their potential roles in plant growth and development were thoroughly explored. Moreover, candidate WOX genes related to the formation of adventitious roots were identified through transcriptomic data and validated using RT-qPCR expression analysis. The results of this research offer significant backing for large-scale genetic engineering breeding initiatives in *P. massoniana*.

## 2. Results

### 2.1. Identification of WOX Genes in P. massoniana

In this research, we utilized BLASTP-HMMER techniques to extract *WOX* genes from the genome of *P. massoniana*, successfully identifying 21 protein sequences of PmWOX. Each member of the PmWOX protein family was subsequently validated using the CCD databases (Table S1) and assigned identifiers from PmWOX1 to PmWOX21. The PmWOX proteins display significant differences in their physical and chemical properties. The lengths of the full-length proteins range from 105 amino acids for PmWOX6 to 482 amino acids for PmWOX4, with molecular weights spanning from 12.43 kDa to 53.15 kDa. The theoretical isoelectric points (pI) vary from 5.42 to 10.01; notably, six of the PmWOX proteins are categorized as acidic, possessing pI values below 7.0, whereas the other 15 proteins are classified as basic. Predictions regarding subcellular localization suggest that all 21 PmWOX proteins are situated within the nucleus.

### 2.2. Classification and Phylogenetic Analysis

**Figure 1:**
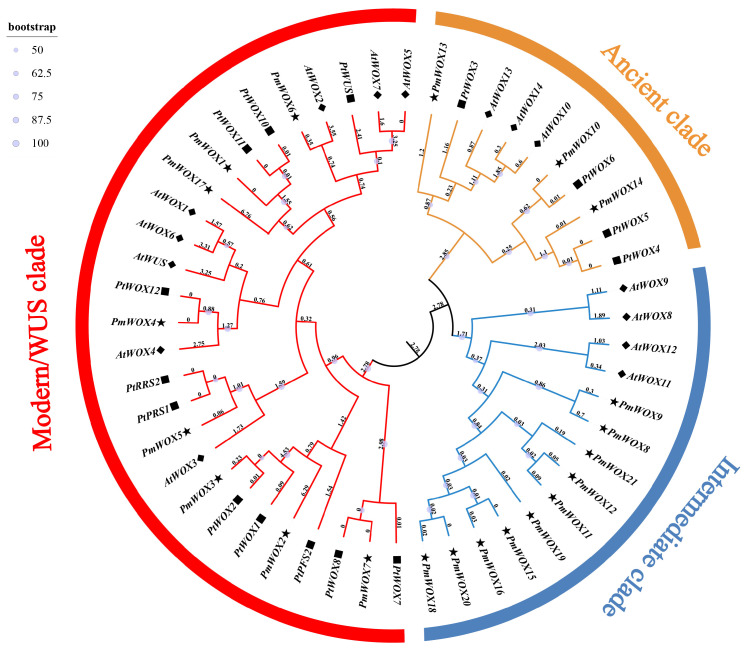
Phylogenetic relationships among WUSCHEL-related homebox (WOX) proteins from *P. massoniana* (Pm), *P. tabuliformis* (Pt), and *A. thaliana* (At). WOX family members were clustered into three groups, with each group displayed in a different color. Labels are omitted on branches with bootstrap values < 50 following standard phylogenetic conventions. Symbols in the tree represent different species: “★” indicate *P. massoniana*, “◆” indicate *A. thaliana*, and “■” indicate *P. tabuliformis*.

To investigate the phylogenetic relationship of PmWOX proteins, a phylogenetic tree was constructed using WOX proteins from three species (*P. massoniana*, *Pinus tabuliformis*, and *Arabidopsis thaliana*). This analysis classified the WOX proteins into three distinct clades: the ancient clade, the intermediate clade, and the modern/WUS clade (Figure 1). The modern/WUS clade comprises seven PmWOX proteins, specifically PmWOX1, PmWOX2, PmWOX3, PmWOX4, PmWOX5, PmWOX6, PmWOX7 and PmWOX17. The ancient clade consists of four PmWOX proteins: PmWOX10, PmWOX13 and PmWOX14. The intermediate clade includes ten PmWOX proteins: PmWOX8, PmWOX9, PmWOX11, PmWOX12, PmWOX15, PmWOX16, PmWOX18, PmWOX19, PmWOX20, and PmWOX21.

To investigate the sequence features of WOX homodomains, we conducted multiple sequence alignments of 51 homodomain amino acid sequences (Figure S1). The distribution of conserved amino acids among the homodomains was strikingly similar across all WOX members from the three species (*P. massoniana*, *P. tabuliformis*, and *A. thaliana*). The study identified that the homodomain comprises several conserved amino acids, including Q and L in helix 1, P, I, and L in helix 2, and N, V, W, F, and N in helix 3.

### 2.3. Chromosomal Location and Collinearity Analysis

**Figure 2:**
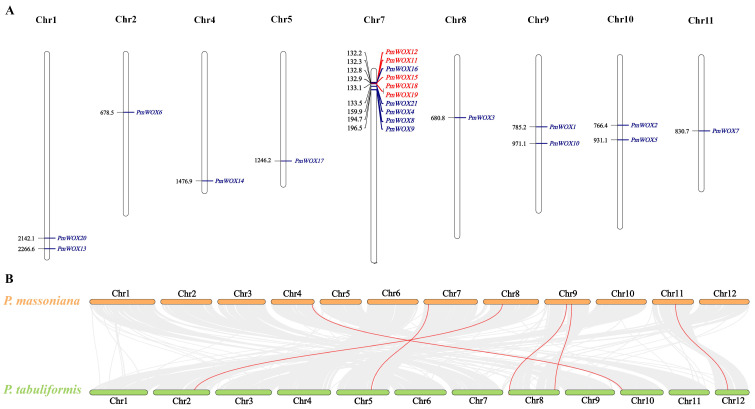
Chromosomal localization and synteny analysis of *PmWOXs.* (**A**) Chromosomal distribution of *PmWOX* genes. The red represents tandem duplication genes. The red color indicates tandem duplicated genes, while blue color indicates other (non-tandem duplicated) WOX genes. (**B**) Collinearity analysis of *P. massoniana* with *P. tabulaeformis WOX* genes. The red line represents the collinear relationship of *WOX*.

Analysis of chromosomal distribution showed that 21 *PmWOX* genes were distributed unevenly across nine chromosomes (Figure 2A). Every chromosome had at least one *PmWOX* gene, with the exception of Chr3, Chr6, and Chr12, highlighting a heterogeneous arrangement. The Chr7 exhibited the greatest number of *PmWOX* genes, totaling 10, while Chr2, Chr4, Chr5, Chr8, and Chr11 each had just one *PmWOX* gene. Additionally, two PmWOX genes were found on each of three chromosomes: Chr1, Chr9, and Chr10.

In the species’ evolutionary background, gene tandem duplication is a key factor in forming gene families. Typically, genes that are situated within 2 kb on the same chromosome are regarded as having emerged from a gene cluster. The analysis of gene duplication in *PmWOXs* revealed the presence of five tandemly duplicated genes, forming two distinct gene clusters (*PmWOX6/7* and *PmWOX9/10/11*) found on Chr7 (Figure 2A). Moreover, when comparing *P. massoniana* with *P. tabulaeformis*, six orthologous gene pairs were identified (Figure 2B), indicating considerable synteny among the *PmWOX* genes.

### 2.4. Conserved Motif and Domain Analysis

**Figure 3:**
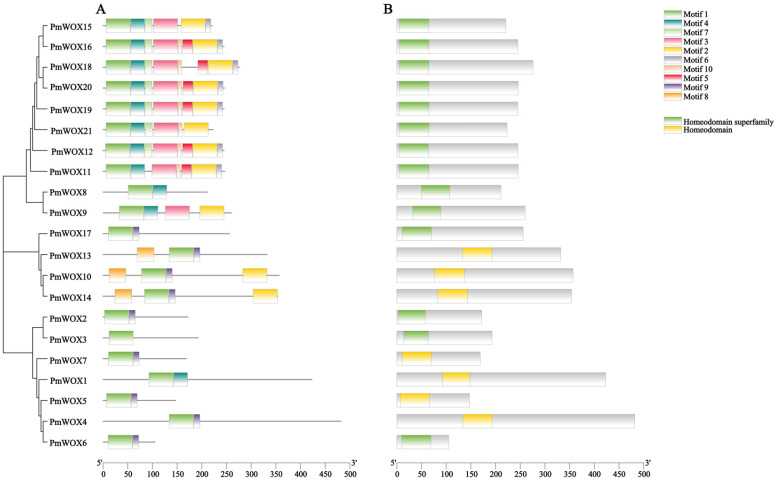
Conserved motif and domain analysis of PmWOX proteins. (**A**) Motifs of PmWOX proteins. Different motifs are distinguished by different colors. (**B**) Conserved domains of PmWOX proteins.

TBtools-II (version 2.458) was employed to visualize the conserved motifs and structural domains of the WOX family members in *P. massoniana*. Motif analysis revealed a total of ten distinct motifs within these PmWOX proteins (Figure 3A). The results indicated that each PmWOX contained between 1 and 8 of the analyzed motifs. Notably, motif 1, which encompasses the WOX family domain, was present in all members. Motifs 3, 5, 6, 7, and 10 were exclusively found in the modern/WUS clade, while motif 8 was unique to the ancient clade, and motif 9 was specific to the intermediate clade. These findings suggest both functional similarities and differences among PmWOX proteins across various subfamilies. The detailed sequences of the different motifs are illustrated in Figure S2. Furthermore, the analysis of conserved domains demonstrated that all PmWOX proteins contained either a Homeodomain superfamily or a Homeodomain domain, with the latter being exclusive to the modern/WUS clade (Figure 3B).

### 2.5. Analysis of Cis-Acting Elements in the PmWOX Promoter

**Figure 4:**
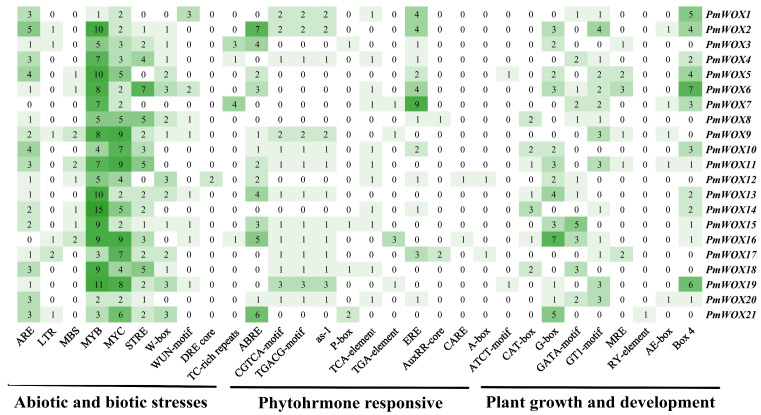
Cis-acting elements analysis of the *PmWOX* genes promoter. Number of major cis-acting elements of *PmWOX* genes. Different colors in the heatmap represent numbers of each cis-acting element.

To comprehensively investigate the possible functions of the *PmWOX* genes, we analyzed the 2-kb regions located upstream of the transcription start site of these genes, utilizing PlantCARE to identify cis-acting elements. We discovered a total of 30 distinct types of cis-acting elements within these genes (Figure S3). The findings revealed a variety of cis-acting elements associated with responses to abiotic and biotic stresses, as well as those linked to phytohormone signaling and the growth and development of the plant within the promoters of *PmWOX* genes (Figure 4). In the realms of abiotic and biotic stress, we identified 10 cis-regulatory elements, the majority of which are responsive to MYB, MYC, and stress-related STRE motifs. Among the elements related to hormone responses, we highlighted the presence of ABRE elements that correspond to ABA responsiveness and ERE elements pertinent to ethylene responses in 13 genes, both demonstrating a notable prevalence. Furthermore, we detected a considerable number of elements tied to MeJA response, including the CGTCA motif and TGACG motif, along with elements relating to the responses of SA and oxidative stress, particularly the activation sequence-1 (as-1)-related elements. In terms of plant growth and developmental processes, the predominant cis-elements comprised G-boxes, GATA motifs, GT1 motifs, and Box4, all of which play a role in responses to light.

### 2.6. Expression Profiles of PmWOX Genes in P. massoniana

**Figure 5:**
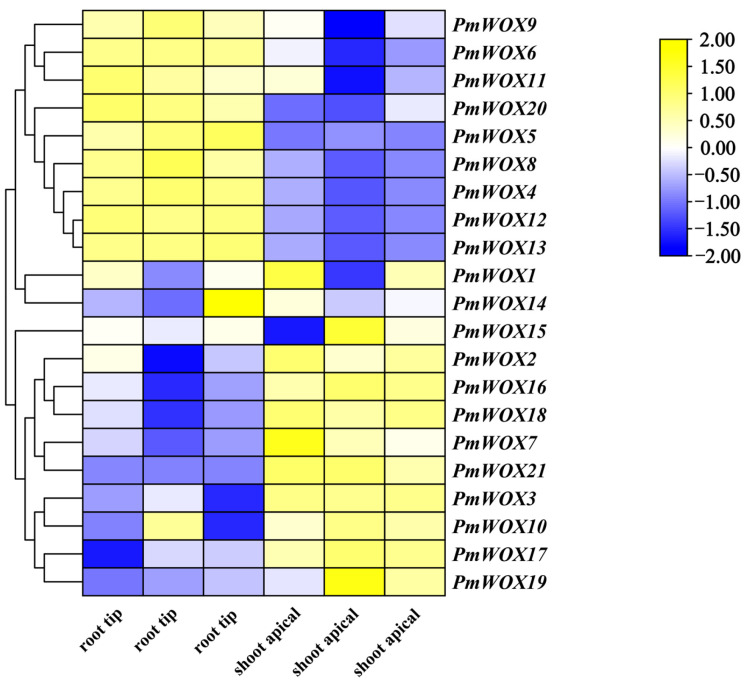
The expression patterns of *PmWOX* genes in *P. massoniana*.

We profiled the expression of PmWOX genes in *P. massoniana* using RNA-seq datasets from root tips and shoot apical meristems. As shown in Figure 5, cluster analysis divided the *PmWOX* gene expression profiles into two major clusters. The first clusters comprised nine genes that exhibited significantly high expression levels in the root tip meristem, while the second consisted of twelve genes that demonstrated markedly elevated expression levels in the shoot tip meristem. Based on the obtained results, we screened nine *WOX* genes with significantly high expression in root tips, namely *PmWOX4*, *PmWOX5*, *PmWOX6*, *PmWOX8*, *PmWOX9*, *PmWOX11*, *PmWOX12*, *PmWOX13* and *PmWOX20*. Since root tips are pivotal tissues for root development, these genes were selected as candidates, and their expression patterns were further validated by RT-qPCR.

### 2.7. Expression Patterns of PmWOX Genes in Different Tissues

**Figure 6:**
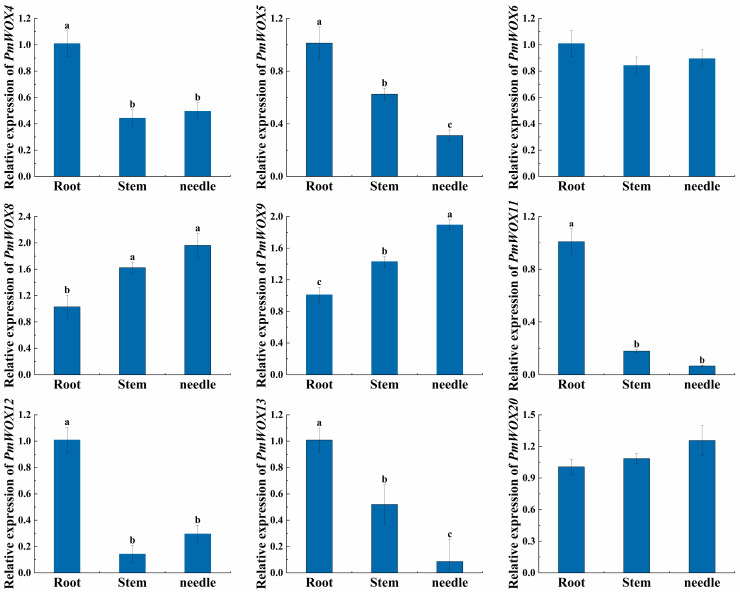
Tissue-specific expression profiles of *PmWOX* genes in *P. massoniana*. The relative expression of each gene was determined in adventitious roots (Root), stems (Stem), and needle-shaped leaves (Needle) via RT-qPCR. Values represent the mean ± SD of three biological replicates. Different lowercase letters denote significant differences among tissues (*p* < 0.05, one-way ANOVA with Duncan’s test).

The tissue-specific expression of nine candidate *PmWOX* genes was examined across adventitious roots, stems, and needles (Figure 6). The *PmWOX* genes displayed divergent expression levels in different tissues. *PmWOX4*, *PmWOX5*, *PmWOX11*, *PmWOX12*, and *PmWOX13* genes reached their maximum expression in the adventitious roots of *P. massoniana*, while *PmWOX8* and *PmWOX9* showed peak expression in needles. In contrast, *PmWOX6* and *PmWOX20* demonstrated no significant differences in expression levels across the various tissues. Based on these findings, the five genes highly expressed in adventitious roots (*PmWOX4*, *PmWOX5*, *PmWOX11*, *PmWOX12*, and *PmWOX13*) were selected for further investigation.

### 2.8. Expression Patterns of PmWOX Genes in Different Root Zones

**Figure 7:**
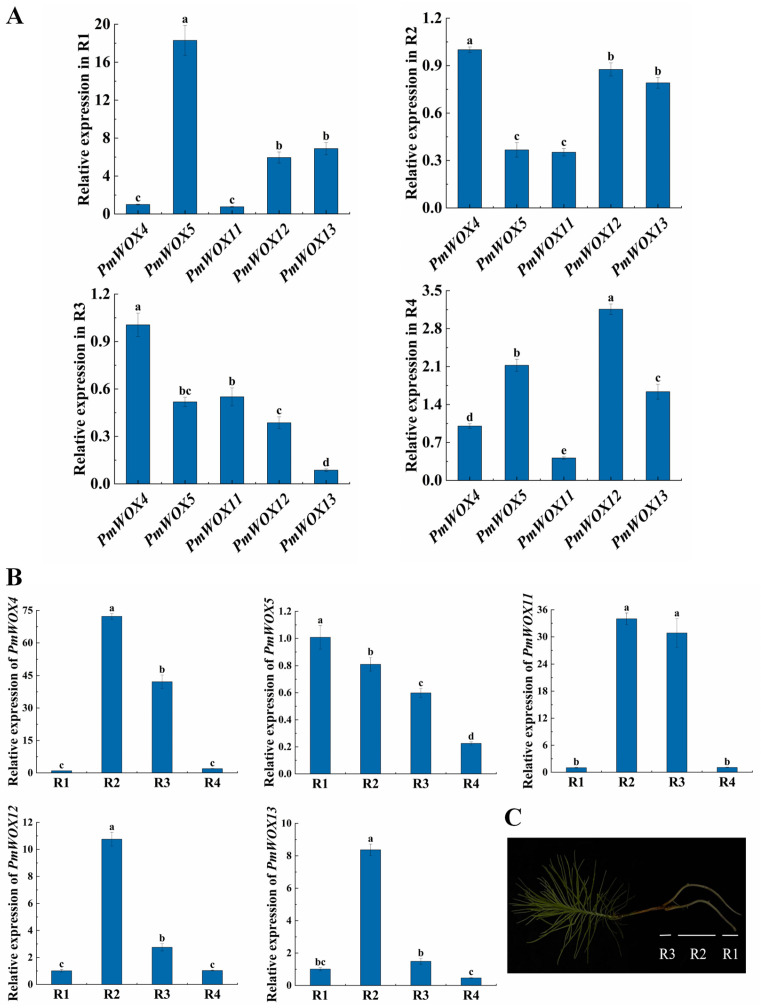
Relative expression level of *PmWOX* gene in different root zones. (**A**) Expression levels of five genes in the same root zones. (**B**) Expression pattern of each individual *PmWOX* gene in different root zones. (**C**) Schematic diagram illustrating the locations of R1, R2 and R3 in adventitious roots. R1: Root tip; R2: Root elongation zone; R3: Root primordium; R4: Lateral root. Values represent the mean ± SD of three biological replicates. Different lowercase letters denote significant differences among tissues (*p* < 0.05, one-way ANOVA with Duncan’s test).

To elucidate expression patterns in different root regions of *P. massoniana*, we examined the relative expression levels of five target genes in four distinct adventitious root zones (Figure 7C) using RT-qPCR. Among the five genes detected in the same root zones, *PmWOX5* exhibited peak expression in root tips; *PmWOX4* was strongly expressed in root primordia and root elongation zones; and *PmWOX12* showed specifically high expression in lateral roots (Figure 7A). For expression variations in each gene in distinct root regions (Figure 7B), with the exception of *PmWOX5*, which exhibited the highest expression level in the root tips, *PmWOX4*, *PmWOX11*, and *PmWOX12* displayed markedly higher expression levels in the root elongation and root primordium than in the root tip zone. Notably, *PmWOX13* showed markedly elevated expression exclusively in the root elongation zone compared with the root tip zone. Although *PmWOX12* and *PmWOX13* also showed detectable expression in root primordia, their expression peaks were located in the root elongation zone, indicating they mainly function in root elongation and morphogenesis. Since adventitious root formation is primarily associated with root primordia development, this result strongly suggests that *PmWOX4* may act as a core regulator during root primordia formation, which is a critical step in adventitious root formation. For this reason, *PmWOX4*, which is significantly highly expressed in root primordia, was screened as a key candidate gene involved in adventitious root formation for subsequent functional research.

### 2.9. Analysis of PmWOX4 Subcellular Localisation, Transcriptional Activity, and Protein Interaction Network

**Figure 8:**
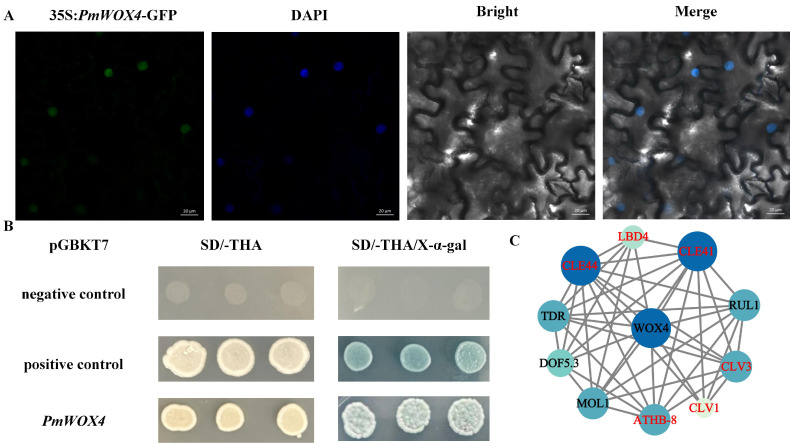
(**A**) The subcellular localization of the *PmWOX4* genes and the empty vector tagged with GFP and transiently expressed in *N. benthamiana*. (**B**) Transcriptional Activity Assay of *PmWOX4* in Yeast. (**C**) Protein interaction network of PmWOX4 protein. The blue circles represent proteins that interact with WOX4, where the color intensity corresponds to the confidence or interaction strength of the prediction. Genes marked in red may interact with PmWOX4 and facilitate adventitious root formation.

To examine the subcellular localization of the PmWOX4, we generated the 35S::*PmWOX4*-GFP fusion expression vector and transiently transformed into *N. benthamiana* leaves via an *Agrobacterium*-mediated infiltration method. Laser confocal microscopy observations revealed that the fluorescence signal of PmWOX4-GFP exclusively localized to the nucleus, which perfectly colocalizing with the DAPI staining (Figure 8A). These data verify that PmWOX4 is a nucleus protein, which is highly consistent with the previous bioinformatics prediction.

To detect the self-activation property of PmWOX4, the *PmWOX4* gene was cloned into the yeast bait plasmid pGBKT7 and introduced into competent yeast cells. Self-activation ability was evaluated based on the growth performance and color reaction of yeast strains on selective defective media. The results showed that both the pGBKT7-*PmWOX4* recombinant strain and the positive control exhibited normal growth on SD/-THA medium (Figure 8B). On X-α-gal containing SD/-THA medium, the two strains grew robustly and produced blue colonies. In contrast, growth of the negative control was inhibited on both selective media. These results demonstrated that *PmWOX4* possesses transcriptional self-activation activity in the yeast system.

With the aid of the STRING database, we generated the PPI network for PmWOX4 (Figure 8C). At present, publicly accessible large-scale PPI data of *P. massoniana* are still scarce, so it is not feasible to build a PPI network directly using protein sequences of this species. In the research of non-model gymnosperms, it is a common and recognized approach to infer conserved interaction networks using homologous proteins from the model plant *A. thaliana*. In *Arabidopsis*, the molecular functions and interaction patterns of *WOX* genes during root growth have been extensively investigated and proven reliable. Here, we first retrieved the orthologous genes of *PmWOX* in *Arabidopsis* through sequence homology comparison. Subsequently, the potential PPI network of PmWOX proteins was predicted based on the validated interaction information of these orthologs, so as to preliminarily dissect the underlying regulatory pathways. The results showed that PmWOX4 shared high sequence similarity with AtWOX4 from *A. thaliana*, suggesting they may have conserved functional characteristics. The predicted PPI network contains multiple functional proteins, including CLE peptide ligands (CLE41, CLE44, CLV3), receptor proteins (TDR, CLV1, MOL1), and transcription factors (LBD4, ATHB-8, DOF5.3, RUL1). Notably, the interaction network includes several regulators widely studied for their functions in vascular stem cell preservation and root organogenesis. For instance, the CLE41-CLE44/WOX4 module is central to cambial activity, and its presence among the predicted partners suggests that PmWOX4 might participate in a similar signaling axis during adventitious root formation. Additionally, ATHB-8, a procambial marker, and LBD4, an inducer of root primordia, further support the functional relevance of these interactions to adventitious rooting. Moreover, the appearance of CLV3/CLV1, although primarily recognized for shoot apical meristem regulation, is noteworthy given emerging evidence that CLV-like signaling also influences the root stem cell niche. Their presence in the network thus hints that PmWOX4 might engage in stem cell homeostasis across different meristem types, which facilitates the adventitious root formation.

## 3. Discussion

As a significant timber species in southern China, *P. massoniana* faces considerable challenges regarding adventitious root regeneration during tissue culture and regeneration processes, which severely restricts the progress of clonal breeding [18]. The availability of genome sequences serves as an invaluable resource for research on *P. massoniana*, providing a critical reference for exploring the genetic basis of functional diversity within this species. The *WOX* transcription factors act as important regulators governing embryogenesis, meristem maintenance, and root regeneration [9,19]. To enhance the genetic traits of root systems in *P. massoniana*, this systematic analysis identified *PmWOX* genes within the *P. massoniana* genome and the key gene *PmWOX4* involved in adventitious root formation was screened out. The present findings provide a framework to uncover the regulatory pathways of *WOX* genes during the growth and development of *P. massoniana*.

WOX family members exert essential functions in developmental processes [20]. To date, a growing number of WOX family members have been discovered in non-model plants. Due to the limited information regarding the *WOX* family in *P. massoniana*, we aimed to identify and characterize *PmWOX* genes through genomic analysis. We screened and identified 21 *PmWOX* genes from the genome sequences of *P. massoniana*. The number of *PmWOX* family members significantly exceeds those found in *P. abies* (11) [16], *Ginkgo biloba* (13) [21] and *P. yunnanensis* (10) [22]. This increase in *PmWOX* family members may be attributed to the extensive genome of *P. massoniana*. Results from phylogenetic clustering indicated that the *WOX* genes are divided into three major clades. This classification aligns with the WOX family classification in species such as *A. thaliana* and *P. tabuliformis*, indicating that *WOX* genes share a highly conserved evolutionary trajectory in both gymnosperms and angiosperms. Members located on one branch of the phylogenetic clade tend to exhibit functional similarities [23]. Except for *PmWOX4*, all other genes from the intermediate evolutionary branch are clustered on chr 7, exhibiting close genetic relationships, suggesting a potential for similar functions. Furthermore, homologs of *AtWOX4* are known to exert important functions in adventitious root formation of woody plants, including apple, walnut, and poplar, implying both functional conservation and divergence [12,13,24]. In the present study, *AtWOX4* and *PmWOX4* were found to be closely related within the modern clade, leading to the hypothesis that *PmWOX4* may exert a similar function in adventitious root formation.

During the process of evolution, both segmental and tandem duplications play a prominent role in expanding the genome [25]. Chromosomal localization analysis showed that *PmWOX* genes are unevenly scattered on nine chromosomes, with chr7 containing 10 genes. The clustered regions exhibited high sequence homology among the genes, confirming that tandem duplication dominates the expansion of the *PmWOX* family. In comparison, we identified six collinear pairs between *P. massoniana* and *P. tabuliformis*, indicating evolutionary conservation at the interspecific level and suggesting that these genes may retain highly stable functions in *P. massoniana*. Conserved motif analysis revealed that motif 1 is present in nearly all WOX protein members, serving as a critical basis for identifying members of the *WOX* gene family. WOX proteins located on one branch of the phylogenetic clade exhibit similar numbers of motifs and identical sequence arrangements, while notable differences in motif counts between different branches may result from the loss or acquisition of conserved motifs during the evolutionary process of WOX family members [26].

Gene promoters contain cis-acting elements that play a crucial role in influencing gene expression patterns by acting as interaction sites for transcription factors [27]. A study examining the cis-acting elements within *PmWOX* genes uncovered various cis-regulatory components linked to hormone responses, development, and stress within the promoter regions. Typically, WOX proteins are regulated by hormones and are involved in fine-tuning hormone signaling pathways that affect numerous biological processes [28]. Of the ten phytohormone-responsive elements found in *PmWOX* genes, the ABRE motif emerged as the most prevalent, orchestrating the biosynthesis of abscisic acid (ABA)—a key pathway vital for enhancing plant resistance [29]. Regarding stress-responsive elements, the frequent occurrence of MYB, MYC, and STRE motifs indicates their involvement in mediating both abiotic and biotic stress responses [30]. Additionally, the identification of plant growth and development elements such as G-box, GATA, GT1 and Box4 suggests their potential involvement in photomorphogenesis [31]. Overall, these results suggest that *PmWOX* genes act as integrative hubs, synchronizing developmental programs with both environmental and hormonal signals.

Transcriptome data analysis revealed that 21 *PmWOX* genes were expressed in both root and shoot apical meristems, a finding that aligns well with known functions of *WOX* family members in meristematic tissues. This suggests that these genes perform essential roles in the apical and root meristems of *P. massoniana*. Further validation through RT-qPCR of nine highly expressed genes in root tips. The results indicated that *PmWOX4*, *PmWOX5*, *PmWOX11*, *PmWOX12*, and *PmWOX13* exhibited significantly high expression in adventitious roots, which points to their involvement in adventitious root formation and growth. Notably, most *PmWOX* genes exhibited distinct expression patterns differed from those of their homologous counterparts in *Arabidopsis* and *populus* [8,23]. To elucidate their functional specificity, we conducted expression analysis in different root regions. Expression analysis revealed that *PmWOX4* displayed high transcript levels in both the root elongation zone and root primordia, implying its potential function in governing adventitious root formation.

In this study, transcriptional activity assays demonstrated that *PmWOX4* exhibits transcriptional self-activation ability. Subcellular localization analysis verified that *PmWOX4* localizes to the nucleus, which is in accordance with bioinformatics predictions and further supports its potential role as a transcriptional regulator. Protein–protein interaction network analysis revealed that PmWOX4 shares high sequence homology with AtWOX4. Previous research has verified that the CLE41/44-WOX4 module acts as the core regulatory pathway for vascular cambium development in *A. thaliana* [32]. The adventitious roots of *P. massoniana* are mainly derived from vascular cambium cells [33]. This predicted interaction suggests that PmWOX4 may participate in the reactivation of vascular-related cells during adventitious root initiation through a similar CLE signaling pathway. In addition, ATHB-8 is involved in procambium specification and vascular development, and adventitious root formation requires the establishment of de novo vascular connections [34,35]. The potential interaction between PmWOX4 and ATHB-8 may synergistically determine the vascular patterning in root primordia. Multiple members of the LBD family in poplar have been confirmed to directly regulate adventitious root formation [36]. As a predicted interacting factor in this study, LBD4 suggests that PmWOX4 may form a transcriptional complex with LBD4 to co-regulate downstream target genes associated with cell wall remodeling or hormone signaling. Recent studies have also demonstrated that CLV signaling sustains the root apical stem cell niche [37]. Its identification in this regulatory network suggests that PmWOX4 may modulate stem cell balance, which in turn facilitates the formation of adventitious root meristems. Collectively, these predicted interactions indicate that PmWOX4 acts as an essential node in the regulatory pathways of vascular and root formation. By integrating multiple signaling pathways including CLE41/44, ATHB-8, LBD, and CLV1/3, PmWOX4 mediates the precise balance between cell proliferation and differentiation during adventitious root formation.

## 4. Materials and Methods

### 4.1. Plant Materials

Seed of *P. massoniana* were gathered from the State-owned Forest Tree Improved Seed Base located in Yu’an District, Lu’an City, Anhui Province, and then sown in the greenhouse at Nanjing Forestry University. The seedlings were cultivated in a nutrient medium that included perlite, vermiculite, and peat mixed in a ratio of 1:1:3, with conditions maintained at a temperature of 25 °C, under a photoperiod comprising 16 h of light and followed by 8 h of darkness. After a period of three months, robust seedlings of *P. massoniana* were chosen for the collection of various tissues and root segments. All samples were promptly frozen using liquid nitrogen and preserved at −80 °C for subsequent analysis. Each experimental treatment was conducted in triplicate, incorporating three independent biological replicates.

### 4.2. Identification of PmWOX Genes

To identify the *PmWOX* genes of *P. massoniana*, the genome was obtained form GigaDB (https://gigadb.org/dataset/102688/, accessed on 15 July 2025) [38]. A Hidden Markov Model (HMM) profile of the WOX family domain (PF00046) was utilized to assign WOX proteins using HMMER 3.0 software [39]. Additionally, we retrieved 15 *A. thaliana* WOX genes as queries for BLASTP-based homology detection. The full catalog of *AtWOX* genes is available in Supplementary Table S3 [40]. Both BLASTP and HMMER analyses were performed using an E ≤ 10^−5^, and sequences with identity above 40% were retained as candidate genes. The results from the HMM and BLASTP search were compared to confirm that all identified sequences contained the WOX protein domain, verified using the CDD (https://www.ncbi.nlm.nih.gov/cdd/, accessed on 18 July 2025). All 21 potential PmWOX proteins were listed in Table S1. Subsequently, ProtParam (https://web.expasy.org/protparam/, accessed on 20 July 2025) was employed to predict the protein length, isoelectric point (pI) and molecular weights (MWs) of all PmWOX proteins, while CELLO v.2.5 (http://cello.life.nctu.edu.tw/, accessed on 20 July 2025) was used to predict their subcellular localization.

### 4.3. Multiple Sequence Alignment and Phylogenetic Analysis

To explore the evolutionary features of the WOX gene family in conifers, we selected *P. tabuliformis* and *A. thaliana* representative species for phylogenetic comparison. The *P. massoniana* and *P. tabuliformis* are typical gymnosperms of Pinaceae with close evolutionary relationships and serve as representative conifer species. As a classic model angiosperm, *A. thaliana* possesses well-annotated *WOX* gene sequences and abundant functional research data. The combination of gymnosperms and the angiosperm model plant establishes a robust comparative framework, enabling comprehensive analysis of the evolution and potential functions of WOX gene families across distinct plant lineages. Subsequently, we retrieved the protein sequences of WOX from *P. tabuliformis* via the CPIR database (http://conifers.cn/, accessed on 25 July 2025). The full list of PtWOX genes is provided in Supplementary Table S3. Multiple sequence alignments were conducted with ClustalX2 utilizing the default settings. The resulting sequences were subsequently adjusted manually with GeneDoc (version 2.6.002). Multiple sequence alignments were trimmed using trimAl v1.5.1 to exclude noisy and ambiguous sites. The maximum-likelihood (ML) phylogenetic tree was constructed with IQ-TREE2 v2.0.7. ModelFinder Plus automatically determined the most appropriate substitution model for amino acid sequences. Branch confidence was measured by 2000 ultrafast bootstrap replicates, and parallel processing was realized with four threads [41]. iTOL (https://itol.embl.de/, accessed on 4 June 2026) was applied for tree visualization.

### 4.4. Conserved Domains and Conserved Motif Analysis

To further understand the function of PmWOX proteins, the sequences of the proteins were analyzed using the MEME program (https://meme-suite.org/meme/, accessed on 18 July 2025) to identify conserved motifs. The settings allowed for zero or one repetition of motifs, with a maximum limit of 10 motifs. The phylogenetic tree, along with the conserved domains and motifs of PmWOX proteins, was visualized through the use of TBtools-II (version 2.458) [42].

### 4.5. Chromosomal Distribution and Synteny Analysis

The localization of all *PmWOX* genes to their respective chromosomes in *P. massoniana* was performed utilizing the gene mapping function provided by the GFF file in Mapchart (Version 7.0). Synteny blocks between the genomes of *P. tabuliformis* and *P. massoniana* were identified through the Quick MCScanX Wrapper and visualized with the Dual Synteny Plotter in TBtools [42,43].

### 4.6. The Cis-Acting Elements in PmWOX Promoter Regions

To pinpoint cis-regulatory elements in *PmWOX* genes, the promoter regions upstream sequence (2 kb) was acquired by aligning the CDS with the genome of *P. massoniana*. An investigation for cis-acting elements was conducted and analyzed with the aid of the online PlantCARE database (http://bioinformatics.psb.ugent.be/webtools/plantcare/html/, accessed on 15 October 2025) and visualized using TBtools [42,44].

### 4.7. Expression Profile Analysis

Based on the transcriptome information from root tips and shoot apical meristem tissues (PRJNA863936), the TPM (transcripts per kilobase million) values for all candidate *PmWOXs* were analyzed utilizing Galaxy (https://usegalaxy.org/, accessed on 28 July 2025). Utilizing TBtools software, a heat map was created reflecting the log2 (TPM + 1) values.

### 4.8. RNA Extraction and Quantitative Real-Time PCR (RT-qPCR) Analysis

Total RNA was harvested from all plant samples with the FastPure Universal Plant Total RNA Isolation Kit (RC411-01, Vazyme Biotech, Nanjing, China). The integrity, concentration and quality of the RNA were evaluated using NanoDrop ND2000 (Thermo Fisher Scientific, Waltham, MA, USA). Following the manufacturer’s instructions for the First Strand cDNA Synthesis Kit (AT311, TransGen Biotech, Beijing, China), 1 µg of total RNA was utilized for cDNA synthesis. The resulting cDNA was tenfold diluted for RT-qPCR and stored at −20 °C. RT-qPCR primers (Table S2) were designed across intron regions with the aid of Primer Premier 5.0 software, using a-tubulin (*TUA*) as the internal control gene. The specificity of the primers was verified through PCR. Each PCR reaction included a 10 µL system, comprising 5 µL of SYBR Green, 1 µL of diluted cDNA, 0.4 µL of each primer and 3.2 µL of ddH_2_O. The thermal cycling program consisted of an initial denaturation at 95 °C for 60 s, followed by denaturation at 95 °C for 15 s, annealing at 60 °C for 15 s, and extension at 72 °C for 10 s, for a total of 40 cycles. Each reaction was conducted in three independent technical replicates. Relative expression levels of all *PmWOX* genes under varying conditions were computed using the 2^−ΔΔCt^ method. Statistical analyses were conducted using IBM SPSS Statistics 26.0. All results are expressed as mean ± standard deviation (SD), and group differences were assessed using ANOVA followed by Duncan’s multiple range test.

### 4.9. Protein Interaction Network and Subcellular Localization Analysis

The STRING database (https://cn.string-db.org/, accessed on 29 October 2025) was used for protein–protein interaction (PPI) network analysis. To determine the subcellular localization of *PmWOX4*, its coding sequence was integrated into the pCAMBIA1302-eGFP expression vector. The recombinant construct was transformed into *Agrobacterium tumefaciens* strain GV3101, and subsequently injected into leaves of *N. benthamiana*. Following a 48-h incubation in darkness, the GFP fluorescence signal was observed using a confocal laser scanning microscope.

### 4.10. Transcriptional Activity Assay

The CDS of *PmWOX4* was integrated into the pGBKT7 vector. Following this, the resultant plasmid was co-transformed into *Saccharomyces cerevisiae* strain AH109 utilizing the LiAc/DNA/PEG technique. In accordance with previously established protocols, transformed yeast cells were first cultured on SD/-Trp medium to identify positive transformants. Subsequently, the samples were transferred to SD/-Trp/-His/-Ade and SD/-Trp/-His/-Ade + X-α-Gal media for transcriptional activation analysis. The empty pGBKT7 vector acted as the negative control, while *PmC3H20*, which has been confirmed to possess self-activation activity, was used as the positive control [45].

## 5. Conclusions

Based on the chromosome-level genome of *P. massoniana*, this study identified 21 members of the *PmWOX* gene family through a genome-wide analysis. Through phylogenetic analysis, we categorized these members into three clades. Analysis of conserved motifs indicated that motif 1 serves as a critical criterion for identifying members of the *WOX* gene family. Additionally, tandem duplication events have been recognized as the main contributors to the growth of the *WOX* gene in *P. massoniana*. Furthermore, cis-element analysis indicated that *WOX* genes are involved in plant growth and may be associated with meristems. Tissue-specific expression analysis demonstrated that nine *PmWOX* genes were predominantly expressed in the root apical region. Moreover, RT-qPCR analysis showed that *PmWOX4* exhibited significantly high expression levels in both the root primordium and the root elongation zone. Further analysis revealed that *PmWOX4* is localized in the nucleus and demonstrates transcriptional activation activity when analyzed in yeast. PPI prediction indicated that PmWOX4 interacts with CLE41/44, LBD, ATHB8 and CLV3/CLV1, thereby contributing to the formation of adventitious roots. Overall, these findings provide a significant groundwork for both the functional characterization and potential applications of *WOX* genes within *P. massoniana*.

## Data Availability

The original contributions presented in this study are included in the article/Supplementary Material. Further inquiries can be directed to the corresponding author.

## Supplementary Materials

The following supporting information can be downloaded at: https://www.mdpi.com/article/10.3390/plants15121845/s1, Figure S1: Multiple sequence alignment analysis of WUSCHEL-related homebox (WOX) proteins from *P. massoniana*, *P. tabuliformis*, and *A. thaliana.* The black blocks indicate several highly conserved residues by the alignment of homodomains in the three species; Figure S2: Identification of conserved protein motifs. Ten distinct conserved motifs were identified using sequence analysis. Each motif is represented listed with its consensus amino acid sequence; Figure S3: Cis-acting elements in the promoters of the *PmWOX* family. Distribution of cis-acting elements in *PmWOX* family promoters. Different colored blocks represent distinct types of cis-acting elements; Table S1: List of the 21 *PmWOX* genes identified in this study; Table S2: Primers used in this study; Table S3: Reference *WOX* genes from *A. thaliana* and *P. tabuliformis* in this study.

## References

[1] Ji K.S., Xu L.A., Wang D.B., Ni Z.X., Wang Z.R. (2022). Progresses and achievements of genetic improvement on Masson pine (*Pinus massoniana*) in China. J. Nanjing For. Univ. (Nat. Sci. Ed.).

[2] Chi Y., Wang G.G., Zhu M., Jin P., Hu Y., Shu P., Wang Z., Fan A., Qian P., Han Y. (2023). Potentially suitable habitat prediction of *Pinus massoniana* Lamb. in China under climate change using Maxent model. Front. For. Glob. Change.

[3] Bai Q., Cai Y., He B., Liu W., Pan Q., Zhang Q. (2019). Core set construction and association analysis of *Pinus massoniana* from Guangdong province in southern China using SLAF-seq. Sci. Rep..

[4] Deng L.X., Chen J.Y. (2008). Study on Genetic improvement and germplasm conservation and utilization of *Pinus massoniana* in Guizhou. J. Anhui Agric. Sci..

[5] Gan D.Y., Lin W., Luo D., Chen J.P., Wang Y. (2022). Comparative afforestation test of tissue-cultured seedlings from different families of *Pinus massoniana*. Hubei Agric. Sci..

[6] Wang Y., Yao R.L. (2019). Increased endogenous indole-3-acetic acid:abscisic acid ratio is a reliable marker of *Pinus massoniana* rejuvenation. Biotech. Histochem..

[7] Zhou X., Han H., Chen J., Han H. (2024). The emerging roles of *WOX* genes in development and stress responses in woody plants. Plant Sci..

[8] Liu B., Wang L., Zhang J., Li J., Zheng H., Chen J., Lu M. (2014). WUSCHEL-related Homeobox genes in *Populus tomentosa*: Diversified expression patterns and a functional similarity in adventitious root formation. BMC Genom..

[9] Yu Y.J., Zhang D.B., Yuan Z. (2016). The updated functional study of WOX protein family in regulating stem cell development. Chin. Bull. Bot..

[10] Zhang X., Zong J., Liu J., Yin J., Zhang D. (2010). Genome-Wide Analysis of *WOX* Gene Family in Rice, Sorghum, Maize, *Arabidopsis* and Poplar. J. Integr. Plant Biol..

[11] Nardmann J., Reisewitz P., Werr W. (2009). Discrete shoot and root stem cell-promoting *WUS/WOX5* functions are an evolutionary innovation of angiosperms. Mol. Biol. Evol..

[12] Xu X.Z., Che Q.Q., Cheng C.X., Yuan Y.B., Wang Y.Z. (2022). Genome-wide identification of *WOX* gene family in apple and a functional analysis of *MdWOX4b* during adventitious root formation. J. Integr. Agric..

[13] Li J., Jia H., Zhang J., Liu B., Hu J., Wang L., Lu M. (2018). Effect of Overexpression of *Populus tomentosa WUSCHEL-related homeobox 4* (*PtoWOX4a*) on the Secondary Growth of Poplar. Sci. Silvae Sin..

[14] Li J., Zhang J., Jia H., Liu B., Sun P., Hu J., Wang L., Lu M. (2018). The *WUSCHEL-related homeobox 5a* (*PtoWOX5a*) is involved in adventitious root development in poplar. Tree Physiol..

[15] Xu M., Xie W., Huang M. (2015). Two WUSCHEL-related HOMEOBOX genes, *PeWOX11a* and *PeWOX11b*, are involved in adventitious root formation of poplar. Physiol. Plant..

[16] Hedman H., Zhu T., von Arnold S., Sohlberg J.J. (2013). Analysis of the *WUSCHEL-RELATED HOMEOBOX* gene family in the conifer *picea abies* reveals extensive conservation as well as dynamic patterns. BMC Plant Biol..

[17] Wang H., Xie Y., Liu W., Tao G., Sun C., Sun X., Zhang S. (2020). Transcription factor *LkWOX4* is involved in adventitious root development in *Larix kaempferi*. Gene.

[18] Wan Y., Fan F. (2024). Direct organ regeneration from apical shoot buds of adult *Pinus massoniana* Lamb. Vitr. Cell. Dev. Biol. Plant.

[19] Hassani S.B., Trontin J.F., Raschke J., Zoglauer K., Rupps A. (2022). Constitutive overexpression of a conifer *WOX2* homolog affects somatic smbryo development in *Pinus pinaster* and promotes somatic embryogenesis and organogenesis in *Arabidopsis* seedlings. Front. Plant Sci..

[20] Li M., Wang R., Liu Z., Wu X., Wang J. (2019). Genome-wide identification and analysis of the *WUSCHEL*-related homeobox (*WOX*) gene family in allotetraploid *Brassica napus* reveals changes in *WOX* genes during polyploidization. BMC Genom..

[21] Xu W., Xu A., Xu P., Li J., Luo C., Yang X., Ming M., Liu Y., Wang G., Xue L. (2025). Transcriptional dynamics and functions of *WUSCHEL-related homeobox (WOX)* genes from *Ginkgo biloba* in tissue culture. BMC Plant Biol..

[22] Xu J., Hu Z., Chen S., Tang J., Chen L., Chen P., Cai N., Xu Y. (2025). Transcriptome-wide identification and characterization of WUSCHEL-related homeobox (*WOX*) gene family in *Pinus yunnanensis*. Plant Biol..

[23] Du F., Chang Z., Kong X., Xia L., Zhou W., Laux T., Zhang L. (2025). Functional conservation and divergence of the *WOX* gene family in regulating meristem activity: From *Arabidopsis* to crops. Plant Physiol..

[24] Chang Y., Song X., Zhang Q., Liu H., Bai Y., Lei X., Pei D. (2020). Genome-Wide identification of *WOX* gene family and expression analysis during rejuvenational rhizogenesis in Walnut (*Juglans regia* L.). Forests.

[25] Lyu Q., Chen S., Wang X., Yuan Y., Zhang H., Liang W., Cheng H., Deng Z. (2026). Genome-Wide identification and expression analysis of the WOX family reveals potential roles in stem development of *Euphorbia hirta*. Plants.

[26] Wang Y., Yang L., Geng W., Cheng R., Zhang H., Zhou H. (2024). Genome-wide prediction and functional analysis of *WOX* genes in blueberry. BMC Genom..

[27] Hajheidari M., Huang S.C. (2022). Elucidating the biology of transcription factor-DNA interaction for accurate identification of cis-regulatory elements. Curr. Opin. Plant Biol..

[28] Dabravolski S.A., Isayenkov S.V. (2025). Exploring hormonal pathways and gene networks in crown root formation under stress conditions: An update. Plants.

[29] Ayub A., Javed T., Nayab A., Nan Y., Xie Y., Hussain S., Shafiq Y., Tian H., Hui J., Gao Y. (2025). *AREB/ABF/ABI5* transcription factors in plant defense: Regulatory cascades and functional diversity. Crit. Rev. Biotechnol..

[30] Yong Y., Zhang Y., Lyu Y. (2019). A MYB-Related transcription factor from *Lilium lancifolium* L. (*LlMYB3*) is involved in Anthocyanin Biosynthesis pathway and enhances multiple abiotic stress tolerance in *Arabidopsis thaliana*. Int. J. Mol. Sci..

[31] Guo X., Li Q., Yan B., Wang Y., Wang S., Xiong F., Zhang C., Zhang Y., Guo L. (2022). Mild shading promotes sesquiterpenoid synthesis and accumulation in Atractylodes lancea by regulating photosynthesis and phytohormones. Sci. Rep..

[32] Hirakawa Y., Kondo Y., Fukuda H. (2010). TDIF peptide signaling regulates vascular stem cell proliferation via the WOX4 homeobox gene in *Arabidopsis*. Plant Cell.

[33] Yao R.L., Wang Y. (2016). Effects of temperature on adventitious root formation of tissue-cultured shoots in *Pinus massoniana*. Guihaia.

[34] Donner T.J., Sherr I., Scarpella E. (2009). Regulation of preprocambial cell State acquisition by auxin signaling in *Arabidopsis* leaves. Development.

[35] Bellini C., Pacurar D.I., Perrone I. (2014). Adventitious Roots and Lateral Roots: Similarities and Differences. Annu. Rev. Plant Biol..

[36] Dang H., Yu C., Nan S., Li Y., Du S., Zhao K., Wang S. (2024). Genome-Wide identification and gene expression networks of LBD transcription factors in *Populus trichocarpa*. BMC Genom..

[37] Hu C., Zhu Y., Cui Y., Cheng K., Liang W., Wei Z., Zhu M., Yin H., Zeng L., Xiao Y. (2018). A group of receptor kinases are essential for CLAVATA signalling to maintain stem cell homeostasis. Nat. Plants.

[38] Chen H., Qin X., Chen Y., Zhang H., Feng Y., Tan J., Chen X., Hu L., Xie J., Xie J. (2025). Chromosome-level genome assembly of *Pinus massoniana* provides insights into conifer adaptive evolution. Gigascience.

[39] Chaudhary R., Singh S., Kaur K., Tiwari S. (2022). Genome-wide identification and expression profiling of WUSCHEL-related homeobox (*WOX*) genes confer their roles in somatic embryogenesis, growth and abiotic stresses in banana. 3 Biotech.

[40] Haecker A., Gross-Hardt R., Geiges B., Sarkar A., Breuninger H., Herrmann M., Laux T. (2004). Expression dynamics of WOX genes mark cell fate decisions during early embryonic patterning in *Arabidopsis thaliana*. Development.

[41] Chen Q.Y., Zhou H.Y., Hu S., Zhou Z.C., Liu B., Gao K., Ji K.S., Liu Q.H. (2026). Identification of ubiquitin genes and their expression patterns in *Pinus massoniana* under infection stress from the pinewood nematode. Plants.

[42] Chen C., Chen H., Zhang Y., Thomas H.R., Frank M.H., He Y., Xia R. (2020). TBtools: An integrative toolkit developed for interactive analyses of big biological data. Mol. Plant.

[43] Wang Y., Tang H., Debarry J.D., Tan X., Li J., Wang X., Lee T.H., Jin H., Marler B., Guo H. (2012). MCScanX: A toolkit for detection and evolutionary analysis of gene synteny and collinearity. Nucleic Acids Res..

[44] Lescot M., Déhais P., Thijs G., Marchal K., Moreau Y., Van de Peer Y., Rouzé P., Rombauts S. (2002). PlantCARE, a database of plant cis-acting regulatory elements and a portal to tools for in silico analysis of promoter sequences. Nucleic Acids Res..

[45] Wang D.B., Yao S., Agassin R.H., Zhang M.Y., Lou X., Huang Z.C., Zhang J.F., Ji K.S. (2022). Transcriptome-Wide identification of CCCH-type zinc finger proteins family in *Pinus massoniana* and RR-TZF proteins in stress response. Genes.

