# Supplementary material for: Genome-Wide Characterization of the WOX Gene Family and Identification of Key PmWOX4 Gene Associated with Adventitious Root Formation in Pinus massoniana
Source: Plants (Basel). 2026 Jun 15;15(12):1845. doi: 10.3390/plants15121845 (PMC13306962; doi:10.3390/plants15121845)
Supplement: Supplementary file 1 [file plants-15-01845-s001.zip › Supplementary Figure.pdf]

Supplementary Figure 1

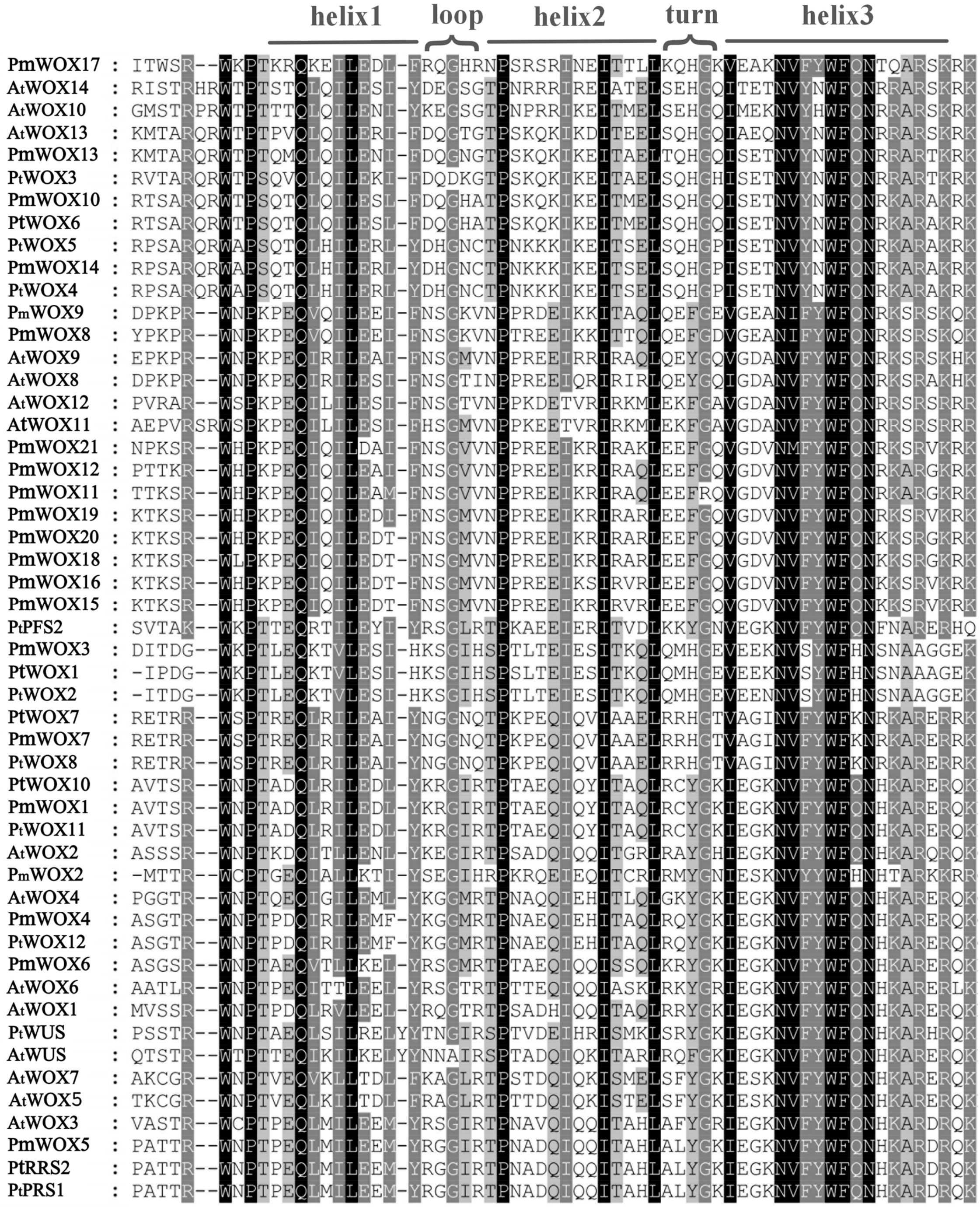

Fig. S1. Multiple sequence alignment analysis of WUSCHEL-related homebox (WOX) proteins from *P. massoniana*, *P. tabuliformis*, and *A. thaliana*. The black blocks indicate several highly conserved residues by the alignment of homodomains in the three species.

Supplementary Figure 2

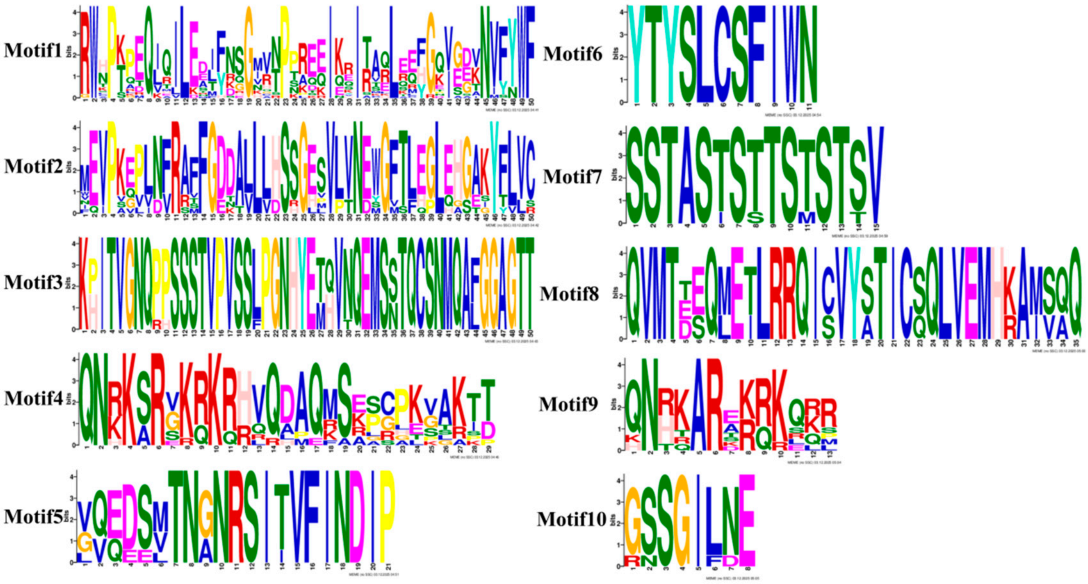

Fig. S2. Identification of conserved protein motifs. Ten distinct conserved motifs were identified using sequence analysis. Each motif is represented listed with its consensus amino acid sequence.

Supplementary Figure 3

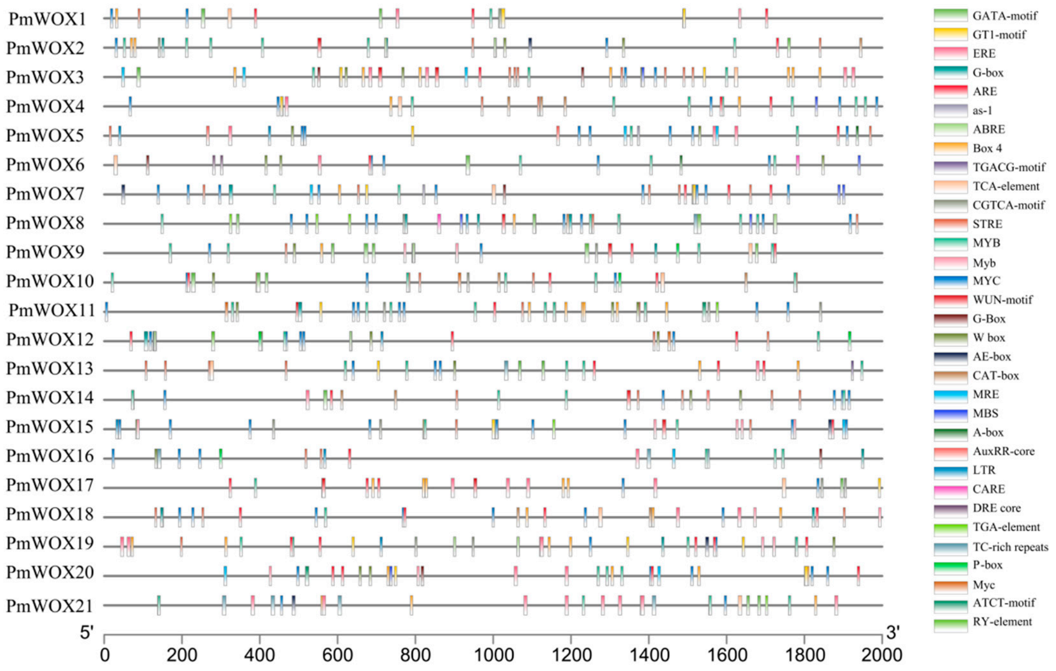

Fig. S3. Cis-acting elements in the promoters of the *PmWOX* family. Distribution of cis-acting elements in PmWOX family promoters. Different colored blocks represent distinct types of cis-acting elements.
